# Editorial Notes

**Published:** 1904-03

**Authors:** 


### Editorial Notes.

**University College Colston Society.**

THE fifth annual meeting of this Society was held on January 21st, 1904, under the presidency of the Right Hon. HENRY HOBHOUSE, M.P., who, in proposing "The Pious Memory of Edward Colston," asked the question how Edward Colston would have spent his money had he been in Bristol to-day. He would have looked round, and would have seen, in the first place, an admirably-organised system of elementary schools, supported by the rates of the city; then he would have seen very excellent secondary schools of every grade and every type, ranging up to their Grammar School and Clifton College. He would next have visited that well-equipped technical institute founded by the Society of Merchant Venturers, where such good work was accomplished in technology and applied science; and last, but not least, he would have cast his eyes upon that which was, or ought to be, the apex of their educational institutions—the University College. That institution, to quote the highest and most impartial authorities, was doing, under able and public-spirited management, the good work of spreading enlightenment and mental culture, of encouraging scientific research, and training with vigour and efficiency professional men and the future teachers of the young; and was doing all this work with inadequate funds and insufficient equipment. It had to do it with no aid from the rates, with a scanty allowance from the spirit duties, and with a most meagre Government grant, doled out by a parsimonious Treasury. There was a constantly-advancing demand for new and better scientific apparatus, more space, and more appliances, and specially, as he now learned, in the departments of electro-technics and bacteriology. Bristol was in some danger of falling behind in higher-grade education—not only behind foreign cities, but many large towns of this country—and he had no doubt the Colston whom he had imagined returning amongst them that day would have devoted a large portion of his fortune to making that University College the acknowledged intellectual centre not only for Bristol but for the West of England. He hoped that citizens, inspired by the noble generosity of Edward Colston, would come forward with funds to raise the efficiency of the College.

The Right Hon. Sir JOHN E. GORST, K.C., M.P., in responding for the guests, spoke on the subject of University Education, and from an experience of seven years in the office of Minister of Education he had come to the conclusion that the country suffered from too many examinations. When they became a University they would have to confer degrees, and he supposed it was impossible to confer these without some kind of examination, but he wished it were possible to make it imperative that all candidates should produce some definite piece of work as a condition for which the degree should be conferred. It was so in many of the older Universities with regard to the awarding of a doctor's degree, where certain definite intellectual work was done and approved, instead of a mere examination, the answering of a number of questions. He did not know whether it was possible for this to be more universally followed. The possession of a University degree should be proof that the holder had done some definite intellectual original work, in recognition of which the distinction had been awarded. Degrees and certificates in some instances had lost their value owing to the small attainments for which they were conferred. There were in America, as they knew, sham universities, which gave sham degrees to people which had no significance at all. There were numbers of people in the world who, without knowing how degrees were acquired, were ready to attach extraordinary value to letters which came after a name, and which in reality meant nothing at all. It had been proposed to take legal notice of this, but he doubted whether they could do anything which would guard them against the folly of mankind. In conferring degrees the greatest possible care should be taken. A degree conferred by a Royal University with a charter from the Crown should be conferred for such solid attainments as would prevent it from being confused with degrees obtained by mere examination. He wished their institution every success when it attained the position which it would as the head of the educational system of the West of England.

The President of Magdalen College, Oxford (Mr. T. Herbert Warren, M.A.), also spoke on the subject of the proposed local University, and said that he believed the granting of degrees by Bristol would before long be a *fait accompli.* He had asked himself upon more than one occasion whether he wished to see Universities multiplying in this country. He had a strong belief in University Colleges. Some seven years ago it was his duty and privilege, representing the Treasury, to visit the University Colleges of Great Britain, and to apply a comparative method to them. They spent a great deal of time and trouble in ascertaining what was really the character of the work being done in those Colleges, and he remembered very well their impression that excellent work was being accomplished. Some of those Colleges had since become Universities, and he was of the opinion that the question of how many should in the future become Universities depended upon the effort that would be made by the localities and communities in which they were situated. They received the impression that the work done in Bristol was of a genuine kind, but they came to the conclusion that there was not sufficient local support. He fought hard for his own city and College, and endeavoured to obtain rather more from the Treasury, but was met with the argument that the local support was insufficient. When he looked at the report sent in about two years ago to the Treasury, by those who had visited the College, he found the tone of that institution was still the same, only even still more emphatic than before, and that the local support had to some extent increased. He thought, however, that it was not yet what it ought to be. Still, a very great deal had been done, and they ought not to be too despondent. They should press forward in the course on which they were well advanced.

Mr. J. W. Arrowsmith, in announcing the financial results of the year, spoke of the scheme for the completion of the

College according to the plan originally laid down, and of erecting a tower in connection with the new wing as a memorial to the late Mr. Albert Fry.

**Recent Honours obtained by University College Students.**

We cordially congratulate the past and present students of the Medical Faculty of the University College, Bristol, who have distinguished themselves and done credit to the Medical School by winning honours of which any School of Medicine may be justly proud. Mr. A. R. Short, M.B., B.Sc. (Lond.), who has had a brilliant student's career, was awarded the Gold Medal and Scholarship, obtaining the first place in Medicine in the Final M.B. Examination, and also First-class Honours in Obstetric Medicine. Mr. J. J. S. Lucas, M.B. (Lond.), F.R.C.S., obtained Honours in Medicine and Forensic Medicine. In the Final B.S. Examination Mr. M. H. Phillips, M.B. (Lond.), F.R.C.S., gained the Gold Medal and Scholarship in Surgery.

**University College. Proposed Athletic Ground.**

In aid of funds to acquire an Athletic Ground for our students, performances of *The Schoolmistress* are announced to take place on April 29th and 30th, in the Victoria Rooms, under the direction of Dr. D. S. Davies. We trust that the efforts being put forth for this most desirable object will meet with the response they deserve, and that friends of the College will interest as many people as possible and get them to support Dr. Davies.

**Royal National Pension Fund for Nurses.**

Attention has recently been called to this fund by a visit from the Secretary, Mr. Louis H. M. Dick, who gave a lecture on the subject in the Board-room of the Bristol Royal Infirmary on January 27th. The matrons, sisters and nurses of various medical institutions were present, and the advantages conferred by the fund were explained. At the conclusion Mr. Joseph Storrs Fry remarked that he and other members of the General Hospital Committee had thoroughly investigated the subject, and were quite satisfied that it was the best thing a nurse could do by way of securing a pension for old age. The valuation of the fund just completed establishes beyond question the fact that the money which a nurse pays into the fund produces a very much better annuity than can be obtained in any other way. The working years of nurses are comparatively few, and some sacrifice in the early years of life becomes a necessity as a provision for the later ones. The committees of various medical charities are doing all they can to assist their nurses in the payment of premiums which must take a comparatively large portion of their yearly income.

**St. John Ambulance Brigade.**

THIS brigade has undertaken a very good work in aid of the National Association for the Prevention of Consumption. A number of men of the local brigade who hold the Nursing Certificate of the Order have been selected to visit at frequent intervals cases of consumption occurring among the poor; and lady helpers have volunteered to do a similar work among those of their sex. The association will supply gratuitously sputum flasks of the Dettweiler pattern to each sufferer, which visitors will leave together with a card of suggestions for the prevention of the disease. By indicating to the sufferer the precautions which should be adopted to prevent re-infection in himself as well as spread of the disease amongst others, and by repeated visitation to see that the suggestions are adopted, it is hoped that the brigade will receive such encouragement as will justify a continuance of this effort.

**Patent Medicines.**

The Workmen's League, Rotherhithe, may be congratulated on their enlightened action in issuing a leaflet to the working classes warning them against paying any attention to the advertisements of quacks and vendors of patent medicines. The leaflet says very truly that "these humbugs prey upon the credulity of the British race," and further on it makes some strong remarks on the action of a certain portion of the press which insert their advertisements. The League considers the subject of sufficient importance to try and bring it under the notice of the Government, and every well-wisher of the people will wish them every success in their endeavours. Surely it is not too much to expect of a Government that can spare time to legislate for the beasts that perish, to pass a short Bill to compel every vendor of a quack medicine to state plainly on each bottle of medicine that is sold the composition of the mixture that it contains, which ought, one would think, to have the effect of making people think twice before taking, for example, iodide of potassium for fancied impurities of the blood, or large doses of nitrate of potassium for imaginary disease of the kidneys. We should like to see this excellent pamphlet more widely circulated, as the working classes are by no means the only offenders in this way, the more educated classes being quite as much, if not more, inclined to pour nasty concoctions, of whose composition they know absolutely nothing, into their ill-used and long-suffering bodies.

**Winsley Sanatorium.**

We are indebted to Dr. Lionel Weatherly for the following information in reference to the sanatorium which is now rapidly being completed at Winsley. A year ago the Committee, who have for some time past been using all their energies to get together sufficient funds to enable them to build and equip the Winsley Sanatorium for the poorer consumptives of the Counties of Somerset, Gloucester and Wilts, and the City and County of Bristol, were much troubled as to the expediency of making a start by building the administrative part of the Sanatorium and some twenty beds as a commencement. They finally decided to recommend this course, and resolutions to that effect were duly passed at the annual meeting held at Bath last spring. On June 4th the foundation stone was laid by Lady Dickson Poynder, and to-day the Committee are able to congratulate themselves on the successful issue of the step they then took. Funds have come in so well that at the present time the whole scheme for the sixty beds is rapidly approaching completion, and the Committee hope to start the good work of this Sanatorium during the early weeks of next October. When the Sanatorium is opened it is intended that it shall be quite up-to-date in all details. There will be forty free beds, and twenty beds at an average cost to the patients of some six shillings per week. The following public bodies, firms, groups of persons in certain towns, and private individuals have given sums for the building of beds and have promised to maintain them:—

| | |
|---|---|
| The Bristol Town Council ... ... ... ... | 20 beds |
| The Bath Town Council ... ... ... ... | 2 ,, |
| The Swindon Town Council ... ... ... | 2 ,, |
| The Gloucester Town Council ... ... ... | 1 ,, |
| The Cirencester Rural District Council ... | 1 ,, |
| The Highworth District Council ... ... | 1 ,, |
| The G.W.R. Swindon Works ... ... ... | 1 ,, |
| The Dean Testimonial Fund, Swindon ... | 1 ,, |
| Messrs. Fry and Sons, Bristol ... ... ... | 1 ,, |
| Mr. John Bright Clark, Street ... ... ... | 1 ,, |
| Mr. and Mrs. W. Clark, Street ... ... ... | 1 ,, |
| Cirencester Town, per Dr. Fowler ... ... | 1 ,, |
| Trowbridge Town, per Dr. Pearse ... ... | 1 ,, |
| Bradford-on-Avon Town, per Dr. Flemming | 1 ,, |
| | 35 beds |

It will be seen that the Committee have now only five beds to be disposed of, and they believe these will very soon be taken up. It cannot be too strongly emphasised that the Committee have determined to give the benefits of this Institution to those parts of the four counties who have helped towards the good result already described. They have appealed in vain to certain districts for help, and the meagre support they have received from various districts must result in these districts finding themselves left without the right of nomination. In actual cash and promises the Committee have received up to December 31st, 1903, the following sums:—

| | | | |
|---|---|---|---|
| In donations, including private beds ... ... | £8,565 | 9 | 2 |
| From Town Councils and District Councils, as per deeds ... ... ... ... ... ... | 6,750 | 0 | 0 |
| Promises from private persons ... ... ... | 450 | 0 | 0 |
| By sale of land ... ... ... ... ... ... | 385 | 4 | 8 |
| By interest and sundries ... ... ... ... | 38 | 5 | 4 |
| | £16,188 | 19 | 2 |

The Committee are organising a Carnival at the Clifton Zoological Gardens during next summer for the purpose of paying for the furniture of the Sanatorium, with Mr. F. E. Metcalfe as their energetic hon. secretary, and they earnestly hope this will result in collecting enough for this purpose. If this result is gained, and the Committee are able to dispose of the other five beds, the balance to be still collected to complete the whole scheme will not be more than some £2,000, which should be readily collected from those districts which so far have not seen fit to hold out the helping hand in this good cause.

We are glad to observe that our late active Secretary for the Wiltshire section is now doing good work in Durban, which has the distinction of being the first town in South Africa to found a branch of the National Association for the Prevention of Consumption.

We regret to learn that the world has lost one of its pioneers in sanatorium treatment, Dr. Dettweiler, late chief physician to the Falkenstein Sanatorium for Consumptives. It was mainly due to his exertions that the movement for providing sanatoria for the poorer classes has been so successfully developed in Germany.

